# Two new combinations in the genus *Distephanus* Cass. (Asteraceae, Vernonieae)

**DOI:** 10.3897/phytokeys.17.4013

**Published:** 2012-09-21

**Authors:** Harold Robinson

**Affiliations:** 1 Department of Botany, MRC 166, National Museum of Natural History, Smithsonian Institution, Washington, DC. 20023-7012

**Keywords:** Compositae, Madagascar

## Abstract

New combinations are provided in *Distephanus* for two species from Madagascar.

## Introduction

In the process of recognizing the proper limits of *Distephanus* Cass. a number of combinations have already been made (e. g., Robinson and Khan 1986) and a number of combinations remain to be done. The following two combinations are needed now because of recent entries in GenBank. Descriptions of both species can be found in Humbert (1960).

## Systematics

***Distephanus ambongensis*** (Humbert) H. Rob., comb. nov. Basionym: *Vernonia ambongensis* Humbert, Bull. Soc. Bot. France 87: 346. 1940.

urn:lsid:ipni.org:names:77122348-1

***Distephanus bara***(Humbert) H. Rob., comb. nov. Basionym: *Vernonia bara* Humbert, Notul. Syst. (Paris) 8: 9–10. 1939.

urn:lsid:ipni.org:names:77122349-1
